# Sustainable Cellulose–Bentonite Composites for Wastewater Treatment

**DOI:** 10.3390/ma18184284

**Published:** 2025-09-12

**Authors:** Faiza Shahzadi, Xiao-Feng Sun, Muhammad Sheraz

**Affiliations:** 1 School of Chemistry and Chemical Engineering, Northwestern Polytechnical University, Xi’an 710129, China; faizashahzadi@mail.nwpu.edu.cn (F.S.); sherazmuhammad@mail.nwpu.edu.cn (M.S.); 2 Shenzhen Research Institute of Northwestern Polytechnical University, Shenzhen 518057, China

**Keywords:** cellulose–bentonite composites (CBC), solution casting, in situ polymerization, electrospinning, adsorption ability, emerging contaminants, heavy metals, dyes

## Abstract

Clean water and uncontaminated soil are fundamental for sustaining life on Earth and are essential for assuring human health, and the use of sustainable adsorption materials has emerged as an effective strategy to reduce the volume of effluents released into the environment. Cellulose–bentonite composites have shown significant promise in water purification due to their high adsorption capacity, structural stability, and eco-friendly nature, making them an effective material for the removal of a wide range of pollutants from contaminated water. The most commonly employed methods of fabrication of cellulose–bentonite composites include solution casting, in situ polymerization, and electrospinning. Wastewater typically contains a variety of toxic contaminants, including synthetic dyes such as Congo red and methylene blue, heavy metals such as Cu, Pb, Hg, Ni, pesticides, and oils. Cellulose–bentonite composites offer an economical and efficient solution for the removal of these pollutants, owing to their synergistic properties—especially when compared to other adsorbents such as activated carbon, nanographene oxide, and metal–organic frameworks (MOFs). However, a systematic evaluation of their fabrication strategies, adsorption mechanisms, and application-related studies remains lacking. Also, there is an urgent need for a comprehensive review that consolidates recent findings on the removal of environmental contaminants and highlights both individual and combined adsorption efficiencies. Therefore, this work focuses on cellulose–bentonite composites as highly promising materials for developing sustainable, high-performance adsorbents tailored for advanced water treatment technologies.

## 1. Introduction

Pollutants in water bodies can come from both natural and human-made sources [1]. Natural sources may originate from microbial activity, geological structures, and naturally existing contaminants in water supplies [2]. The majority of pollutants are man-made and result from human activities aimed at enhancing quality of life through industrialization. Heavy metals (including lead, mercury, and cadmium), synthetic colors, and unused drugs are among pollutants that are added in the environment by humans [3]. They also include insecticides [4], herbicides [5], volatile organic compounds (VOCs) [6], and hydrocarbons made from oil. All pollutants pose a threat to human health, as they are associated with diseases, including respiratory issues and cancer [2]. Numerous methods are available for removing pollutants from water, including physical, chemical, and biological approaches [7]. Each method has its own advantages and disadvantages and the choice of method primarily depends on the type and extent of contamination, cost, and efficiency [8]. Natural adsorption materials, such as cellulose and bentonite, have been studied for their ability to remove pollutants.

**Figure 1:**
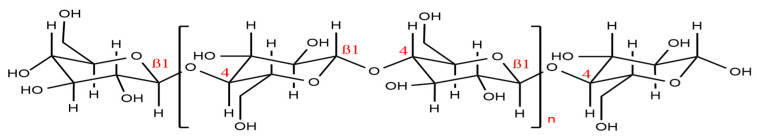
Chemical structure of cellulose with β (1 → 4) glycosidic linkages between glucose units, where β1 indicates the beta configuration at carbon 1, 4 denotes the C4 position of the adjacent glucose, and n represents the repeating number of units in the polymer chain.

Cellulose is a natural, linear polysaccharide consisting of β-D-glucose 1 [9] monomers interconnected by β (1 → 4)-glycosidic linkages with the chemical formula (C_6_H_10_O_5_)_n_ [10,11]. The chemical structural formula of cellulose is shown in Figure 1. It is the most abundant organic polymer found on Earth and serves as the primary structural component of plant cell walls [12,13]. Cellulose is commonly found in nature, and its high molecular weight and unbranched chain, with repeating glucose units, make it a prominent material [14]. It is present in the cell walls of bacteria and fungi, making it available for versatile applications. Cellulose is eco-friendly, biodegradable, and sustainable [15,16,17]. However, its insolubility presents challenges for its use, requiring modifications into forms such as cellulose composites and cellulose nanocrystals [18,19]. The low thermal resistance of cellulose, along with its high susceptibility to microbial attack, further limits its practical applications [20]. With its strong, interconnected network of fibers held together by hydrogen bonds, cellulose exhibits high tensile strength—a property that makes it highly suitable for composite preparation [21]. For all cellulose-based composites in which cellulose acts as a matrix and the other component acts as a reinforcement [22], the combined role of the matrix and the reinforcement enhances the mechanical performance and interfacial compatibility of cellulose and further expands its applications in other fields. This makes cellulose a promising material for numerous applications in electronics [23], packaging [24], construction, and the removal of environmental pollutants [25]. Among various modifications of cellulose such as cellulose nanofibers [26], bacterial cellulose [27], and nanocrystals [28], the development of cellulose-based composites has attracted considerable interest due to their improved mechanical strength [29], adsorption capacity [30], and functional versatility [31]. These composites can be categorized based on the nature of the integrated material, including cellulose–polymer composites (CPC), cellulose–inorganic composites (CIC), cellulose–metal composites (CMC), cellulose–carbon-based composites (CCC), cellulose-based hydrogels (CH), cellulose films (CF), and cellulose membrane composites (CMC) [32,33].

**Figure 2:**
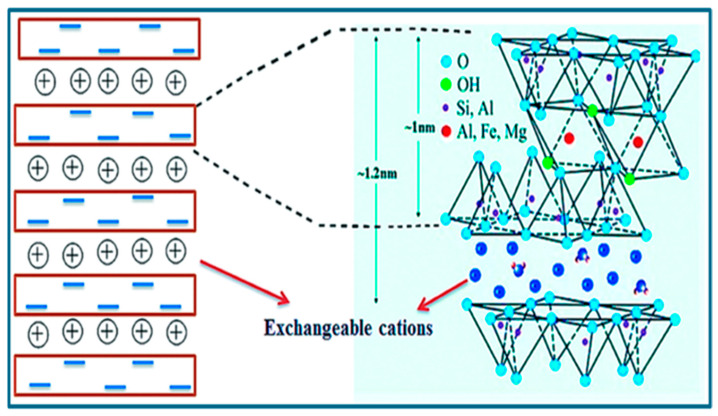
Crystal structure of bentonite clay illustrating negatively charged layers with interlayer exchangeable cations (**left**) and the atomic arrangement of tetrahedral (Si, Al) and octahedral (Al, Fe, Mg) sheets with interlayer spacing (~1.2 nm) (**right**) [41]. Copyright: image has been taken with permission from @ 2014 RSC Advances.

Bentonite is a naturally formed clay primarily consisting of montmorillonite, a swelling clay mineral that is produced when volcanic ash is altered [34]. Depending on the dominant exchangeable cations, there are two kinds of bentonite clays, sodium bentonite and calcium bentonite [35]. Both sodium and calcium bentonite are high-swelling clays but bentonite clay with a higher sodium content tends to have a higher affinity for water molecules than calcium bentonite, as monovalent Na^+^ ions create strong hydration shells, which facilitate extensive interlayer expansion and allow the clay to imbibe large amounts of water [36]. This low cost mineral is located in Earth’s crust, exhibiting favorable mechanical properties [37], thermal stability [38], drug entrapment efficiency [39], sustained release characteristics, and cation-exchange capacity [40]. These unique qualities have made it very useful in many areas, especially for adsorbing and eliminating toxins from water bodies. Figure 2 shows the structural formula of bentonite with exchangeable ions, which help in absorption of different kinds of emerging contaminants [41]. Na^+^ and Ca^2+^ are exchangeable interlayer cations located between the clay layers, where they facilitate adsorption through cation exchange processes [42]. Due to its high water absorption and colloidal qualities, bentonite is useful as an adsorbent and has various industrial applications [43].

Bentonite itself is prone to agglomerating in a water system, lowering the effective surface area and limiting its efficiency in adsorption [44]. In general, bentonite has low mechanical strength [45]. It also lacks high selectivity, demonstrates low affinity toward organic pollutants, and shows limited overall performance [46]. To overcome this, it is typically combined with materials like cellulose to enhance its stability, selectivity, and reusability [47]. Cellulose–bentonite composites have better structural stability, adsorption efficiency, and compatibility with the environment. Cellulose has hydrophilic functional groups (–OH, –COOH) that can form hydrogen bond, attract ions, and exchange ions with bentonite. This creates a reliable hybrid network that can be used to clean water. The composite is strong enough to hold up, can be recycled, and can be made into several practical shapes. Thus, significant efforts have been made for the development of different types of cellulose–bentonite composites that can be used in the removal of pollutants from the environment. Several bentonite–polymer composites, including cellulose-based systems, have been developed for targeted adsorption and separation applications. Although there are diverse adsorbent materials, such as biochar, activated carbon [48], sponges [49], carbon nanotubes [50], metal-layered oxides, and metal–organic frameworks (MOFs) [51], they suffer from several drawbacks when compared with cellulose–bentonite composites. Pristine forms of these materials generally exhibit lower adsorption capacities than their hybrid or functionalized counterparts, highlighting the need for surface modification or particle incorporation to achieve satisfactory results. However, such modifications often increase fabrication complexity and cost [52]. Another major disadvantage is poor regeneration ability, and chemical regeneration violates the structural integrity of adsorbents like sponges or activated carbon and generates secondary effluents that must undergo further treatment, while thermal regeneration is not suitable for metal-based adsorbents due to alterations in their structure and physicochemical properties. These restrictions reduce long-term reusability [48,53]. On the other hand, cellulose–bentonite composites are eco-friendly and can be modified to remove a variety of pollutants. Shamshudin prepared a cost-efficient bentonite–zeolite–acrylic-polymer-supported adsorbent coating (Ben-Zeo-Acry), which has been employed for the removal of cationic (antiseptic) dye [54]. A ferromagnetic bentonite/magnetite cellulose hybrid film effective for adsorbing dyes and heavy metal ions was synthesized [55]. A cellulose acetate–bentonite mixed matrix membrane was designed to separate and selectively remove carbon dioxide (CO_2_) from gas mixtures such as CO_2_/CH_4_ and CO_2_/N_2_ [56]. A chitosan/carboxymethyl cellulose/bentonite/CuO nanocomposite demonstrates effective removal of pathogenic bacteria and organic dyes, serving dual roles as an antibacterial and photocatalytic agent in wastewater treatment [57]. A sodium carboxymethyl cellulose/polyvinylpyrrolidone bentonite composite with Soluplus was formulated as a self-adhesive patch to enhance the dermal absorption of poorly water-soluble β-glycyrrhetinic acid, and the effects of bentonite shape and size on formulation effectiveness was also studied [58].

The environment is increasingly contaminated by emerging pollutants, and there is a pressing need to remove them in a cost-effective and efficient manner [59]. Wastewater treatment has garnered significant attention due to the presence of hazardous contaminants such as heavy metals, dyes, and pesticides [53]. Cellulose–bentonite composites, which combine bentonite’s high surface area and ion exchange capacity with cellulose’s abundant functional groups and structural stability, serve as an efficient adsorbent for removing environmental pollutants, including heavy metals, dyes, and pharmaceutical residues [54]. Studies have shown that cellulose–bentonite composites exhibit a notable adsorption performance for heavy metals, comparable to or even exceeding that of cellulose or bentonite individually [60,61]. In this review, we describe the details of cellulose–bentonite composites, including preparation methods, adsorption mechanisms, and applications of cellulose–bentonite composites in our environment to remove emerging contaminants such as toxic dyes, heavy metal ions, pesticides, and oil.

## 2. Preparation Methods of Cellulose–Bentonite Composites

Table 1 lists preparation methods of cellulose–bentonite composites [62,63,64,65,66,67,68,69]. The fabrication of cellulose–bentonite composites was classified into physical methods and chemical methods. Chemical methods include grafting, in situ polymerization [70], surface functionalization [71], and hydrothermal treatment, while physical methods include solution casting, mechanical dispersion, blending [70,72], electrospinning [73], and layer-by-layer assembly [74]. Solution casting, electrospinning, and in situ polymerization are discussed in detail in the following sections.

### 2.1. Solution Casting

**Figure 3:**
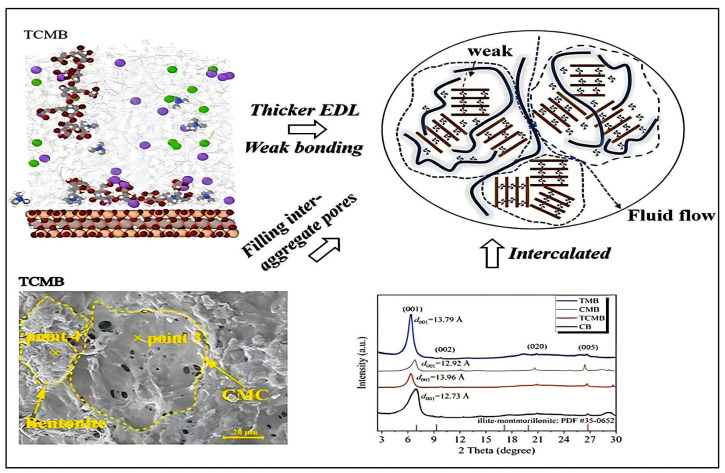
Structural intercalation and morphological characteristics of CMC–bentonite composite prepared via solution casting method [75]. Copyright: image has been taken with permission from @ 2024, Elsevier.

A popular method for creating composite films is solution casting by mechanical dispersion, especially for polymer–clay systems like cellulose–bentonite composites. This process is simple, economical, and suitable for creating thin films with evenly distributed fillers [75]. With the addition of microcrystalline cellulose and nano-bentonite hybrids, thermoplastic starch hybrid bio-composite films were created as a sustainable substitute for traditional non-degradable plastics [76]. Alekseeva et al. [77] developed flexible ethyl cellulose/bentonite composite films with bentonite loadings ranging from 0 to 5 wt%. The resulting films exhibited a uniform structure and enhanced mechanical stability due to the effective dispersion of bentonite within the ethyl cellulose matrix. The final films had a consistent thickness ranging from 0.01 to 0.03 mm, suitable for various environmental and functional applications. Wang et al. [78] developed a cellulose-based hydrogel by using a low temperature NaOH/urea aqueous solution to dissolve cellulose, followed by the addition of exfoliated bentonite (BT) and an optional cross-linker (BDE). The homogeneous mixture was cast into molds and gelled at 60 °C. The formed hydrogel was neutralized and subsequently immersed in LiCl solution to impart ionic conductivity. This method combines solution casting, physical crosslinking via Al–O–C coordination, and ionic doping using LiCl. This method is generally used for the manufacturing of multifunctional composites, including biomedical substances and absorbents [35]. Ni et al. [79] studied structural features of carboxymethylcellulose CMC–bentonite, as depicted in Figure 3, offering a number of practical benefits. It was found that improved compatibility and interaction at the polymer–clay interface was suggested by the intercalation of CMC chains into the bentonite gaps, as shown by the increased d_001_ spacing in the XRD (D8 PHASER X) pattern. Because the aligned clay platelets create a tortuous path that prevents fluid and gas permeation, this intercalated configuration helps to improve barrier properties. Furthermore, the SEM (EM-3010) micrograph shows that CMC efficiently fills the bentonite structure’s interaggregate pores, producing a more compact morphology with fewer voids, as shown in Figure 3. The film’s dimensional stability and mechanical integrity can be greatly enhanced by this kind of structural densification.

### 2.2. Electrospinning

**Figure 4:**
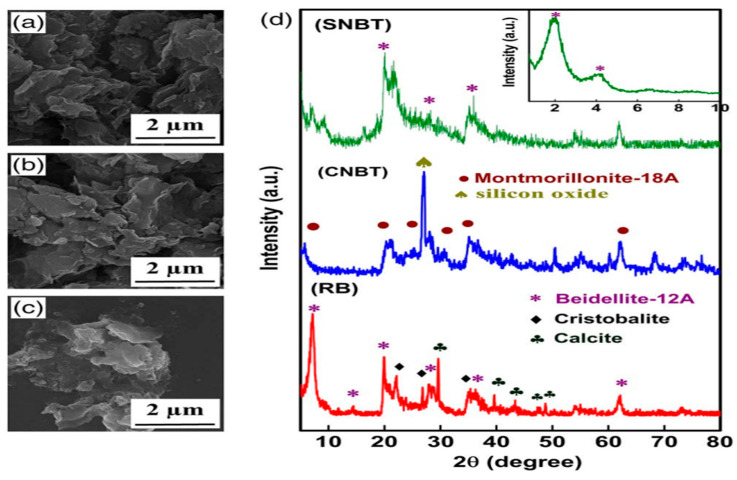
SEM micrographs of (**a**) raw bentonite (RB), (**b**) cellulose–natural bentonite composite (CNBT), and (**c**) cellulose–sodium bentonite composite (SNBT) illustrating the layered morphology of clay particles and their integration within the fibrous cellulose network formed by electrospinning. (**d**) XRD patterns of RB, CNBT, and SNBT showing the presence of characteristic phases [73]. Copyright from Wiley 2020.

Zeaiean et al. [73] synthesized nanobentonite-poly(vinyl alcohol)-bacterial cellulose nanocomposites via electrospinning, mainly for wound-healing purposes. The mixture was electrospun under optimized voltage, flow rate, and tip-to-collector distance to produce continuous nanofibrous mats. The resulting mats were dried and characterized for structural, mechanical, and antibacterial properties. Figure 4 shows the SEM images of the cellulose–bentonite composites prepared by the electrospinning method. Tsekova et al. [80] studied fibrous cellulose acetate/nanoclay (CA/NC) composites that were prepared via the electrospinning method. Initially, CA was dissolved in an acetone/water mixture (80:20 *v*/*v*) at a concentration of 10 wt%, after which nanoclays (10 wt% relative to CA) were integrated into the spinning solution. The resulting CA/NC mixtures were homogenized through vigorous stirring and sonicated to ensure uniform dispersion of the fillers. Electrospinning was then carried out using a syringe pump system at a constant feed rate, under a high voltage of 25 kV, with a tip-to-collector distance of 15 cm and a rotating collector to obtain fibrous mats. Finally, the spun composites were vacuum-dried at 30 °C to remove residual solvents, yielding well-structured fibrous CA/NC composites suitable for water treatment applications. Figure 4 shows the SEM (Philips 515, Tokyo, Japan) images of the cellulose–bentonite composites prepared by the electrospinning method. Bazbouz et al. [65] prepared cellulose–bentonite nanofibers through a free surface electrospinning technique. In this method, natural bentonite clay was first purified using hydrochloric acid and subsequently activated using sodium carbonate to obtain sodium bentonite. To ensure stable dispersion, carboxymethyl cellulose was incorporated into the system. Cellulose acetate was dissolved in an acetic acid water solution and blended with varying concentrations of the activated bentonite. The resulting mixture was electrospun at high voltage using a wire electrode configuration, producing nanofibres with bentonite encapsulated in jellyfish-like semi-spherical structures. An optimum bentonite loading of 5–10 wt% was reported, while higher concentrations led to poor dispersion and impaired fiber formation. Tsekova et al. [80] prepared cellulose–bentonite composite (CBC) nanofibers using the electrospinning technique via blending cellulose acetate (CA) with purified bentonite clay (BC), in which cellulose and bentonite clay were cross linked with and without the presence of glutaraldehyde. The resulting composites were characterized by Fourier transform infrared spectroscopy, X-ray diffraction, thermogravimetric analysis, and differential scanning calorimetry. The percentage yield achieved was greater for the polymer blend made with glutaraldehyde as the crosslinker. BC was first purified and chemically modified, then dispersed in CA solution prepared in a mixture of acetic acid and water. This mixture was electrospun using a needleless setup based on a wire electrode setup to generate multiple jets of polymer solution under a high voltage electric field. The resultant fibers dispersed BC particles within the CA matrix, forming a homogeneous composite nanofiber mat with enhanced mechanical and improved filtration efficiency.

### 2.3. In Situ Polymerization

**Figure 5:**
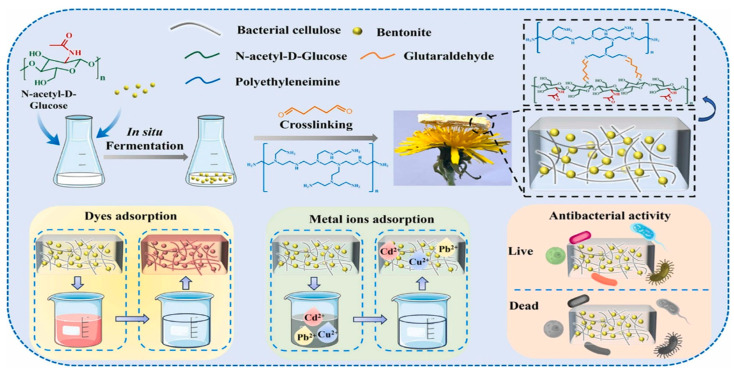
Illustration of the fabrication of bacterial cellulose–bentonite composites via in situ polymerization. Bacterial cellulose is produced through in situ fermentation, followed by the incorporation of bentonite and crosslinking with polyethyleneimine and glutaraldehyde. The composite demonstrates efficient removal of dyes and metal ions, as well as antibacterial activity [81]. Copyright: images have been taken with permission from @ 2024, Elsevier.

Modification of cellulose and bentonite can be carried out using an in situ method by fermentation, which has many advantages, such as simple operation, high efficiency, and avoiding use of hazardous substances. Zhao et al. [81] developed bacterial cellulose–bentonite @polyethylenimine (BCB@PEI) composite membranes in situ. The culture medium comprised N-acetyl-D-glucosamine, yeast extract, disodium phosphate anhydrous, xanthan gum, and K_2_NO_3_. Bentonite was incorporated into the BC culture medium, with N-acetyl-D-glucosamine as the carbon source. The suspension was fully mixed with an electric blender to achieve optimal dispersion of bentonite particles in the culture media. The bacterial suspension was then introduced into the fermentation medium. Following static fermentation for a period of one day, the resulting membrane was designated as BCB, which indicates the presence of amide bonds and also encapsulation of bentonite within the bacterial cellulose membrane. Figure 5 shows the in situ polymerization of bacterial cellulose–bentonite @polyethylenimine and its use in dye adsorption and metal adsorption.

Wang et al. [67] developed a bacterial cellulose/inorganic gel composite of bentonite (BC/IGB) that was efficiently synthesized via an in situ method in both HS medium and corncob hydrolysate. The composites synthesized in these two media exhibited improved water retention capacities. The compositing process was dependent on the modifying environment, with the culture medium playing a significant role in sugar utilization and BC yield. Compared to the ex situ process, the in situ-synthesized BC/IGB composite showed a higher water-holding capacity.

## 3. Adsorption Mechanism of Cellulose–Bentonite Composites

**Figure 6:**
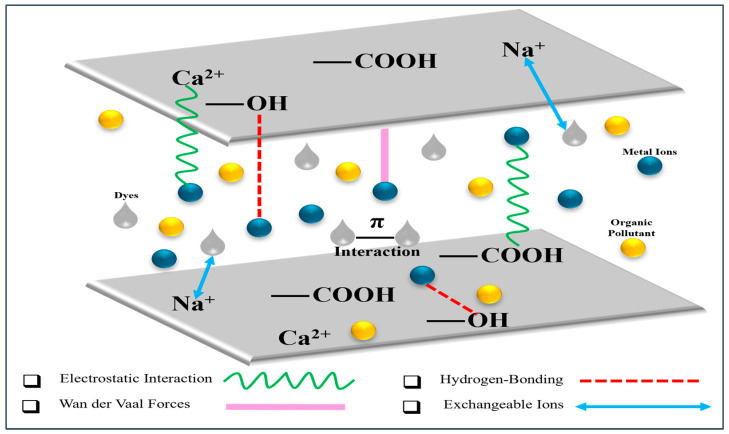
Schematic diagram of adsorption mechanism of cellulose–bentonite composites, illustrating the interactions of heavy metal ions (blue), oil molecules (yellow), and dye molecules (gray) with surface functional groups (–COOH, –OH) on the cellulose–bentonite composite layer through ion exchange and coordination processes.

Both physical and chemical processes are involved in the adsorption mechanism of cellulose–bentonite composites as a result of the combination of cellulose and bentonite. Benitoite has a high cation-exchange capacity by virtue of its layered aluminosilicate structure, with Na^+^ and Ca^2+^ ions in the interlayers being replaced by heavy metal ions such as Pb^2+^, Cd^2+^, and Cu^2+^. At the same time, the abundance of hydroxyl (–OH) and carboxyl (–COOH) groups in cellulose aids in electrostatic attraction and hydrogen bonding with cationic dyes as well as polar organic impurities. Moreover, π-π interactions occur between aromatic dye molecules and the functional groups of the composites, along with weaker van der Waals forces stabilizing neutral organic impurities adsorption. Collectively, they facilitate cellulose–bentonite composites to adsorb a wide range of pollutants like heavy metals, dyes, and organic contaminants efficiently, thereby demonstrating enhanced adsorption performance compared to their individual constituents. Figure 6 shows a schematic diagram of the adsorption mechanism of cellulose–bentonite composites.

Many factors influence the adsorption behavior between the adsorbent and the pollutant [77], such as the properties of the adsorbents. Cellulose–bentonite composites have been very effective and eco-friendly adsorbents for the elimination of a variety of environmental pollutants due to their combined structural and functional properties [34]. Bentonite, being a layered aluminosilicate clay, has a high surface area and cation-exchange capacity, and it is possible to intercalate and adsorb metal ions and organic molecules through electrostatic attraction and ion exchange mechanisms [82]. Upon mixing with nanocellulose, particularly carboxylated or sulfonated varieties, the composite gains additional functional groups (e.g., –OH, –COOH, –SO_3_^−^) which facilitate surface complexation and hydrogen bonding with impurities such as heavy metals, dyes, and drugs [83]. Cellulose incorporation enhances bentonite layer dispersion, mechanical stability, and reusability [84]. Also, the synergic interaction between active surface sites of cellulose and interlayer sites of the clay increases the accessibility and number of centers of adsorption, thereby enhancing the efficiency of removal [85].

**Figure 7:**
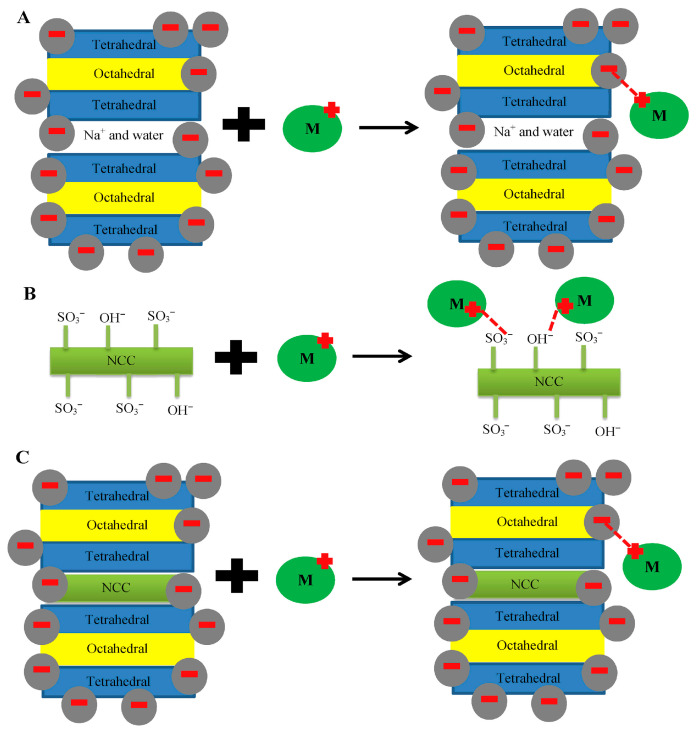
Schematic illustration of adsorption mechanisms for metal ion (M^+^) removal using (**A**) layered clay minerals, (**B**) nanocrystalline cellulose (NCC), and (**C**) NCC-intercalated clay composites [86]. Copyright: images have been taken with permission from @ 2017, Elsevier.

Putro et al. [86] proposed adsorption mechanisms of heavy metals onto bentonite, nanocrystalline cellulose (NCC), and their composite, which are predominantly governed by electrostatic interactions. Bentonite possessed a negatively charged layered structure, which favors adsorption of metal ions onto its surface and interlayer space. NCC, possessing hydroxyl and sulfonate groups, also enables metal ion adsorption by electrostatic attraction and surface complexation. NCC–bentonite composites couple these mechanisms, presumably synergizing the adsorption capacity via the cooperative action of clay layers and functionalized cellulose. Figure 7 shows the mechanism of adsorption for metal ion removal using layered clay minerals, nanocrystalline cellulose (NCC), and NCC-intercalated clay composites. Langmuir and Freundlich models were used to determine the adsorption characteristics of the cellulose–bentonite composite membrane in the adsorption of the pollutants [87]. The Langmuir model supposes that a monolayer is created on the uniform surface by the adsorbate and that there are lower intermolecular interaction forces between adsorbate molecules compared to adsorbate molecules and the surface [88]. The Freundlich model, on the other hand, is predominantly employed to characterize non-ideal or multilayer adsorption, based on the concept that the adsorptive ability of the material diminishes as the surface concentration increases [86,89].

## 4. Applications in Removing Emerging Contaminants

Nowadays, wastewater treatment is in high demand due to the global shortage of clean water. Moreover, it enables the recovery of minerals and chemicals through recycling, not only conserving water resources but also facilitating the reuse of valuable materials [90]. Harmful chemicals in water cause serious health issues if they are consumed by human beings and other living organisms [91,92]. Cellulose–bentonite composites are a next-generation bio-nanocomposite material that offers a green and effective solution for the removal of emerging environmental contaminants [34]. Such contaminants include dyes, pesticides [89], heavy metals [90], oil [91], pharmaceuticals [92], and nano-plastics [93], which are not efficiently removed by conventional wastewater treatment facilities but can be effectively adsorbed using cellulose–bentonite composites [94,95].

### 4.1. Removal of Dyes

**Figure 8:**
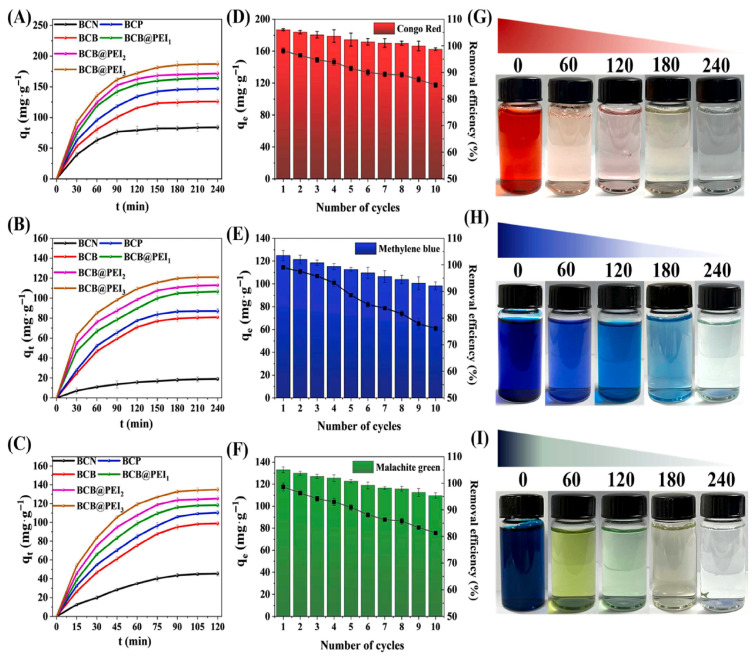
Adsorption of (**A**) Congo red, (**B**) Methylene blue, and (**C**) malachite green dyes by BCN, BCP, BCB, BCB@PEI_1_, BCB@PEI_2_, and BCN@PEI_3_. (**D**–**F**): recyclability of BCB@PEI3 membrane. (**G–I**): photographs of color variation of Congo red, Methylene blue, and malachite green dyes adsorbed by BCB@PEI3 composite membranes over different time intervals (0, 60, 120, 180, 240 min) [81]. Copyright: images have been taken with permission from @ 2024, Elsevier.

Dyes enter water systems primarily through industrial and domestic activities, with the largest contributions coming from the textile, paper, leather, plastic, and printing industries, where dyes are extensively used for coloration. During manufacturing and processing, a significant portion of these dyes do not bind to the product and are discharged as colored wastewater. Adsorption is the best way to remove these dyes from water systems. Azha et al. [93] studied an acrylic polymer–bentonite composite coated on cotton cellulosic fiber (APS/Ben–CCF) to efficiently remove cationic methylene blue (MB) dye. Structural and compositional characterizations by XRD, SEM, and UV–Vis confirmed the successful formation of the composites and coating. APS/Ben-CCF exhibited excellent adsorption performance in a wide pH range, with 100% MB removal within 2 h at 50 ppm. The adsorption was endothermic and adsorption capacity increased at higher temperatures. The obtained results show that APS/Ben-CCF is an easily separable, reusable, and fast-acting cationic dye adsorbent for removing cationic dyes. Zhao et al. [81] prepared a bacterial cellulose–bentonite@polyethylenimine composite membrane, and it could decolorize water through the synergy of electrostatic interaction, where charged dye molecules were attracted to oppositely charged surface sites of the membrane; physical adsorption, where the porous structure and high surface area of the membrane provided by bacterial cellulose and bentonite facilitated the adsorption of dye molecules; hydrogen bonding, where hydroxyl and amide groups of the BC component interacted by bonding, in the form of hydrogen bonds, with dye molecules; and complexation, where some of the dye molecules were capable of forming complexes with functional groups on the surface of the membrane, synergistically acting to enhance the effectiveness of dye removal. Figure 8 shows that the BCB@PEI3 composite membrane has excellent long term application prospects for adsorption of organic dyes. It combines the advantages of bentonite and PEI with greater adsorption capacity for Congo red (186.97 mg g^−1^), methylene blue (121.09 mg g^−1^), and malachite green (134.82 mg g^−1^). After 10 cycles of repeated adsorption processes, the removal rate of the three dyes decreased slightly, but it demonstrated that active adsorption sites on the membrane surface progressively became saturated and the BCB@PEI3 composite membranes still had significant adsorption capacities. These results confirm that PEI functionalization is highly beneficial for anionic dye removal, whereas unmodified BCB is more effective for cationic dyes, with all composites displaying satisfactory long-term reusability.

**Figure 9:**
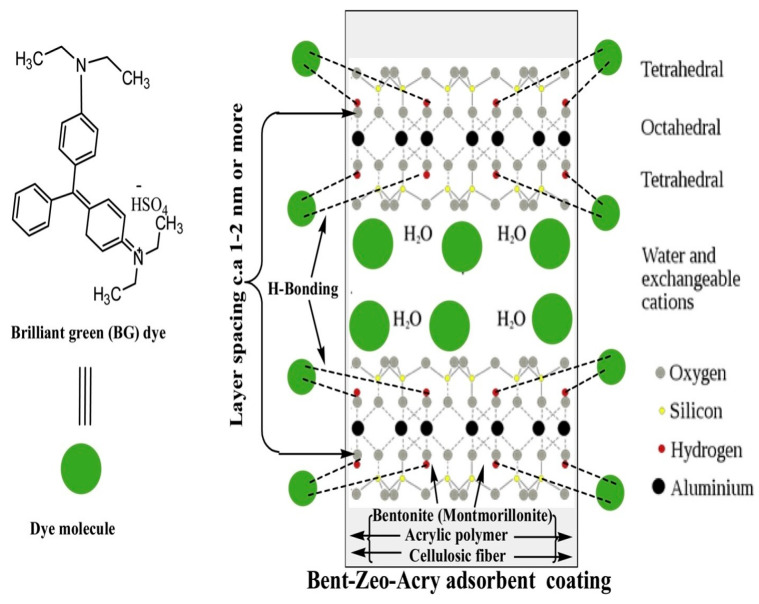
Schematic illustration of the adsorption mechanism of brilliant green (BG) dye molecules onto a Bent-Zeo-Acry composite adsorbent composed of bentonite (montmorillonite), zeolite, acrylic polymer, and cellulosic fibers. The diagram highlights the interaction between dye molecules and the composite layers through hydrogen bonding within the tetrahedral–octahedral–tetrahedral clay structure and the zeolitic framework. The expanded interlayer spacing, water molecules, and exchangeable cations facilitate enhanced adsorption capacity. Elemental representations (oxygen, silicon, hydrogen, aluminum) are shown to visualize molecular interactions at the nanoscale [54]. Copyright: image has been taken with permission from @ 2019, Elsevier.

Santoso et al. [94] prepared cellulose–bentonite (CB) porous composite hydrogels for adsorptive removal of Congo red (CR), an anionic azo dye, from aqueous solutions. The CB hydrogels, synthesized by the incorporation of bentonite clay into a cellulose hydrogel matrix, possess enhanced adsorption potential compared to pure cellulose hydrogels. Physisorption is the dominant process in the adsorption, and the equilibrium data are well-described by the Langmuir isotherm model, indicating monolayer adsorption. The CB hydrogels are also exhibited to be promising as soilless culture media for plant crops like *Vigna radiata* L. and *Arabidopsis thaliana*, indicating their eco-friendly nature and multi-functionality. Shamsudin et al. [54] discuss the removal of dyes, e.g., brilliant green (BG), from water using an affordable bentonite–zeolite–acrylic polymer-supported coating (Ben-Zeo-Acry) adsorbent, as shown in Figure 9, and they synthesized the Bent-Zeo-Acry composite adsorbent by mixing bentonite, zeolite, and acrylic polymer and coating cotton strips with them. Adsorption took place via dye molecule interaction with the zeolite surface and interlayer pores, with montmorillonite (bentonite) present in the coating. The coating performance was influenced by conditions such as the bentonite-to-zeolite ratio, dye concentration, temperature, and pH. The Ben-Zeo-Acry coating demonstrated a high removal efficiency BG from aqueous solutions and therefore shows promise as a good material for wastewater treatment.

Pan et al. [95] developed a novel and very efficient method for the removal of dyes from wastewater by using aminated cellulose/montmorillonite mesoporous composite beads (ACeMt) derived from bagasse cellulose. ACeMt beads, synthesized with the addition of a pore forming agent to enhance porosity, exhibit superior adsorption capacities for cationic and anionic dyes, particularly Auramine O, in comparison with conventional adsorbents. The parameters of pH and temperature influence the adsorption process, while the beads were found to be reusable through desorption and regeneration cycles, making ACeMt a highly efficient, eco-friendly, and versatile bio-adsorbent for water clarification. The dye removal occurs through electrostatic interactions, where oppositely charged dye molecules and surface functional groups attract; hydrogen bonding, where –OH, –NH_2_, and –COOH groups form directional bonds with dye molecules; and Van der Waals forces, providing weak nonspecific attractions and π–π stacking, where aromatic rings in dyes align with conjugated structures on the membrane surface to enhance binding. Together, these interactions synergistically improve adsorption efficiency for diverse dye types. Table 2 lists the removal efficiencies of different dyes by cellulose–bentonite composites, pure cellulose, and pure bentonite.

### 4.2. Removal of Metals

**Figure 10:**
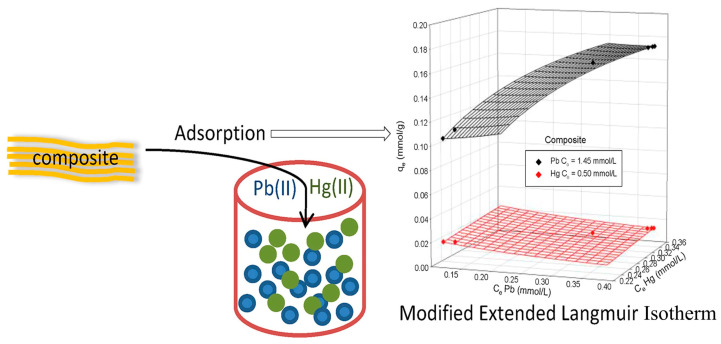
Illustration of Pb^2+^ and Hg^2+^ ion adsorption by a composite adsorbent material, accompanied by a 3D plot based on the modified extended Langmuir isotherm model. The diagram shows competitive adsorption behavior with initial concentrations of Pb^2+^ at 1.45 mmol/L and Hg^2+^ at 0.50 mmol/L. The surface fitting indicates higher adsorption affinity for Pb^2+^, as reflected by the higher adsorption capacity (qe), confirming the composite’s selectivity and efficiency for heavy metal removal [86]. Copyright: image has been taken with permission from @ 2017, Elsevier.

Heavy metals, such as lead (Pb), mercury (Hg), cadmium (Cd), and arsenic (As), are persistent environmental contaminants with serious risks to ecosystems and human health [113]. Heavy metals can accumulate in soil, water, and organisms due to industrial activities, mining, improper disposal of waste, and the use of agrochemicals [114]. Heavy metals are harmful even at low concentrations and are capable of disrupting biological processes, leading to neurological, kidney, and developmental disorders in humans [115]. Their non-biodegradability makes them particularly hazardous as they can remain in the environment for decades [116]. Effective removal of heavy metals from soils and waters is therefore of critical significance. Techniques such as adsorption by nanocomposites, membranes, or bio-materials are increasingly being explored for cost-effectiveness, efficiency, and environmental friendliness in heavy metal pollution remediation [117]. Putro et al. [86] investigated the application of nanocrystalline cellulose (NCC) and bentonite nanocomposite in both single and binary systems. The composite matrix combines the advantages of both components: NCC provides enhanced adsorption capacity through its functional groups, while bentonite contributes its layered structure and cation-exchange capacity. The extended Langmuir model suitably described the binary adsorption isotherms having competitive adsorption of Pb^2+^ and Hg^2+^ on the composite material. The nanocomposite acts as a good potential adsorbent for the removal of heavy metal from contaminated water, with greater favorability of adsorption for Pb^2+^ than for Hg^2+^. Figure 10 is an illustration of Pb^2+^ and Hg^2+^ ion adsorption by the composite adsorbent material, accompanied by a 3D plot based on the modified extended Langmuir isotherm model.

**Figure 11:**
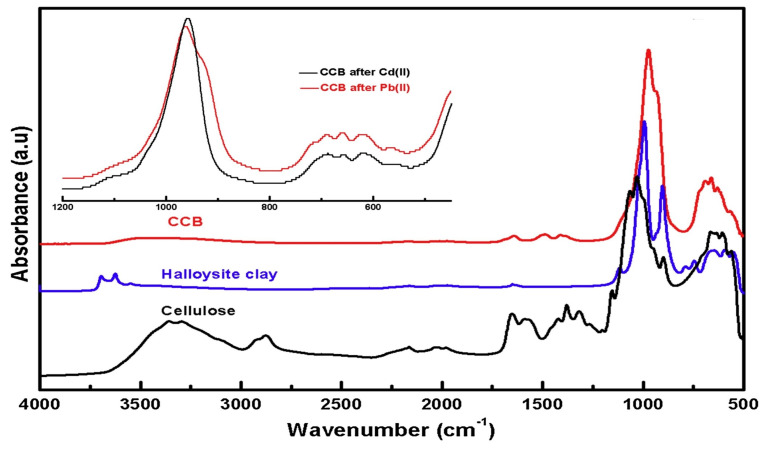
FTIR spectra comparing cellulose, halloysite clay, and the CCB composite before and after adsorption of Cd^2+^ and Pb^2+^ ions. The spectral shifts and intensity variations, particularly in the 1000–500 cm^−1^ region, indicate successful interactions between the composite’s functional groups and the heavy metal ions. The inset further highlights the distinct spectral changes post-adsorption, confirming the effective binding of Cd^2+^ and Pb^2+^ to the CCB surface [118]. Copyright: image has been taken with permission from @ 2020, Elsevier.

Abu Danso et al. [118] synthesized a clay–cellulose biocomposite (CCB) which effectively adsorbed Pb^2+^ and Cd^2+^ ions from aqueous solutions through electrostatic attraction, surface complexation, and ion exchange. With a negative surface charge achieved through NaOH pretreatment, the CCB adsorbed positively charged metal ions and benefitted from a high surface area and porosity, providing abundant adsorption sites. Batch and fixed-bed column studies demonstrated that adsorption was favored at pH > 4, was more efficient at higher adsorbent dosages, and followed rapid kinetics described by the pseudo-second-order model. Further, the CCB could be recycled after regeneration with 0.1 M NaOH as eluent, demonstrating its potential as a good and environmentally friendly adsorbent for heavy metal removal during wastewater treatment operations. Figure 11 shows FTIR spectra of the CCB composite before and after adsorption of Cd^2+^ and Pb^2+^ ions.

Li et al. [119] studied a carboxymethyl cellulose sodium/bentonite composite (CMCMW) adsorbent to remove Cd^2+^ from wastewater through several mechanisms. The modified CMC provided complexation sites by -COOH and -OH groups, while bentonite provided electrostatic attraction due to its negatively charged surface. The composite material also contained Na^+^ ions, hence favoring cation exchange, and a porous structure that enabled pore fixation of Cd^2+^. The microwave-assisted synthesis enhanced the interaction of CMC and bentonite to create a stable framework with abundant adsorption sites for the efficient removal of Cd^2+^. Hokkanen et al. [120] studied hydroxyapatite-bentonite clay nanocellulose (CHA-BENT-NCC) composite to effectively remove cadmium (Cd^2+^) and nickel (Ni^2+^) from water through a synergy of mechanisms: ion exchange, where metal ions are exchanged with calcium ions in the hydroxyapatite crystalline structure and calcium/magnesium ions in bentonite clay; dissolution-precipitation, where dissolution of hydroxyapatite is followed by precipitation of insoluble metal phosphates; and adsorption onto bentonite surfaces, particularly under acidic conditions, where ionized silanol and aluminol sites are the sites of metal binding. Chen et al. [121] developed an adsorbent, bentonite-chitosan-microcrystalline cellulose aerogel (BT-CS-MCCA), synthesized via a bidirectional regeneration strategy, for effective removal of Pb^2+^ ions from polluted water. The BT-CS-MCCA incorporated the benefits of chitosan (high surface area and amino groups), microcrystalline cellulose (mechanical strength and stability), and bentonite (enhanced rate of adsorption). The material was found to have a high adsorption capacity of 256.24 mg g^−1^ and achieved adsorption equilibrium in 60 min, offering a cost-effective and environmentally friendly solution for treating Pb^+2^-contaminated water.

Hokkanen et al. [122] investigated a composite material, CHA-BENT-NCC, for the removal of arsenic (III) from water by leveraging the presence of the adsorption properties of its components. More than 95% removal of As^3+^ takes place in 5 min and is pH-dependent in the range of 4 to 7. Characterization techniques confirmed the success of the synthesis and material morphology. Adsorption kinetics follow a pseudo-first-order model, best described by the Langmuir isotherm. Physical adsorption via functional group interactions on the surface of CHA-BENT-NCC explained the removal process. Luo et al. [85] researched the adsorption of Pb^2+^ from water using magnetic cellulose nanocomposite beads (MCNB) made of carboxyl-functionalized magnetite nanoparticles and acid-activated bentonite (AAB). The MCNB were synthesized using an extrusion dropping technology, which blended cellulose with MN-CA and AAB in NaOH/urea aqueous solution. The process was influenced by contact time, initial heavy metal ion concentration, adsorption isotherms, and solution pH. The process was feasible, spontaneous, endothermic, and mainly controlled by chemical processes like complexation, ion exchange, and electrostatic forces. The beads could be regenerated using sodium citrate, and they would still have their original loading capacities, showing their potential for water decontamination. Table 3 lists the removal efficiency of different metals ions by cellulose–bentonite composites, pure cellulose, and pure bentonite.

### 4.3. Removal of Pesticides

**Figure 12:**
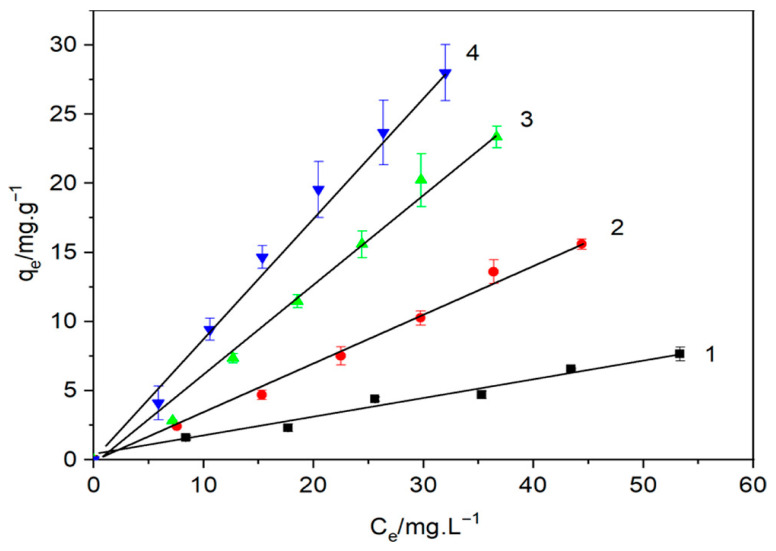
Isotherms for alachlor adsorption on cationic cellulose modified bentonite: (1) bentonite, (2) CC_5_B, (3) CC_10_B, and (4) CC_20_B [140]. Copyright: image has been taken with permission from @ 2022, American Chemical Society.

Pesticides are widely used in agriculture for pest control and optimization of crop yields, but their uncontrolled and indiscriminate use has led to extensive surface and groundwater contamination. The chemicals, including organophosphates, carbamates, and chlorinated hydrocarbons, are typically persistent, toxic, and capable of harming aquatic ecosystems at trace concentrations [137]. Once introduced into water bodies through agricultural runoff, leaching, or improper disposal, pesticides may inflict major health risks on humans and animals owing to their carcinogenic potential, endocrine disrupting efficacy, and neurotoxicity. In view of their chemical stability and low biodegradability, conventional water treatment methods are inadequate for their complete removal, thus novel advanced adsorbent materials must be devised [138]. Bio-polymer composites such as cellulose–bentonite composites have also been introduced as prospective contenders due to their high surface area, tunable surface chemistry, and ability to interact with a broad range of pesticide molecules through hydrogen bonding, electrostatic interactions, and hydrophobic associations. Recent advancements in nanocomposite hydrogel systems have demonstrated a great potential for adsorption and controlled release of pesticides from aqueous solutions [139]. Wang et al. [140] synthesized a novel nanocomposite hydrogel through the mixture of cationic cellulose (CC), modified bentonite (B), and sodium alginate (SA) for the removal of alachlor. Cationic cellulose was synthesized through quaternization of cellulose which comprises positive charges that enhance electrostatic interactions with pesticide molecules having negative charges. Altered bentonite, characterized by a high adsorption capacity and surface area, was incorporated to maximize the activity of the hydrogel. The gel matrix was sodium alginate, which contributed structural integrity and biocompatibility. The study tested a range of formulations, CC_10_B_2_._5_-SA, CC_10_B_10_-SA, and CC_10_B_30_-SA, to determine the impact of bentonite concentration on the hydrogel properties. Results indicated that increasing concentrations of bentonite led to a denser network structure, enhancing the adsorption capacity of the hydrogel for the model herbicide Alachlor. Adsorption isotherm results revealed that the maximum adsorption capacity was for the CC_10_B_30_-SA formulation due to the synergistic effect of cationic cellulose and bentonite. Permeability experiments also revealed that a higher bentonite content reduced the permeability of the hydrogel, suggesting a more compact structure is conducive to extended pesticide release. Figure 12 shows isotherms for alachlor adsorption on cationic cellulose-modified bentonite: (1) bentonite, (2) CC_5_B, (3) CC_10_B, and (4) CC_20_B.

### 4.4. Removal of Oil

**Figure 13:**
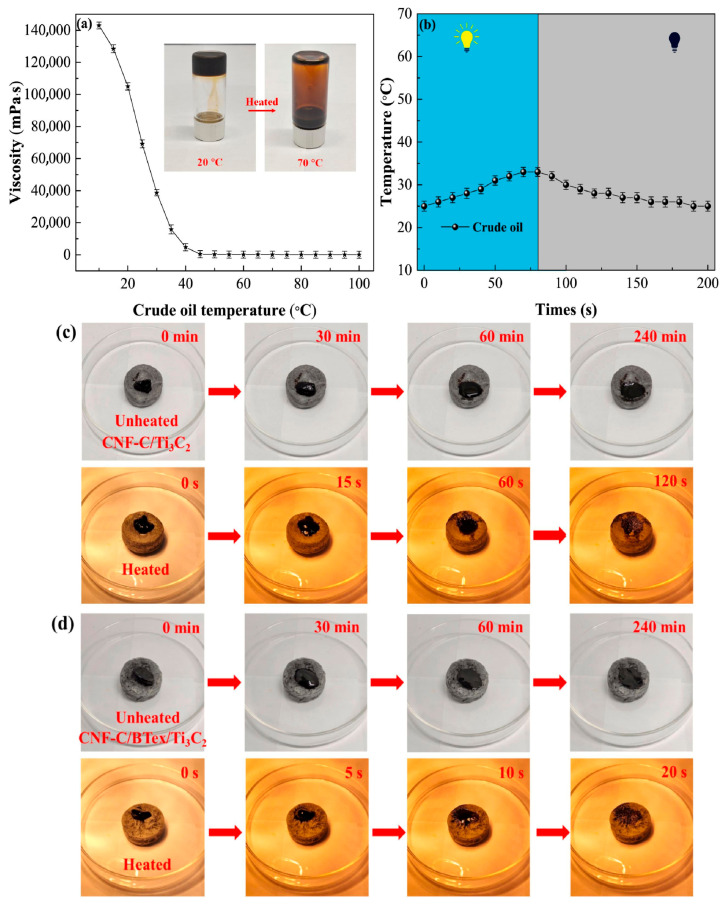
Photographic demonstration and quantitative analysis of adsorption performance of cellulose nanofiber-based composites. The figure (**a**,**b**) shows temperature- and time-dependent viscosity of crude oil, and the figure (**c**,**d**) shows time-dependent removal of crude oil from aqueous solutions using CNF–CTi3C2 and CNF–C/BTex/Ti3C2 under unheated and heated condition [142]. Copyright: image has been taken with permission from @ 2023, Elsevier.

**Figure 14:**
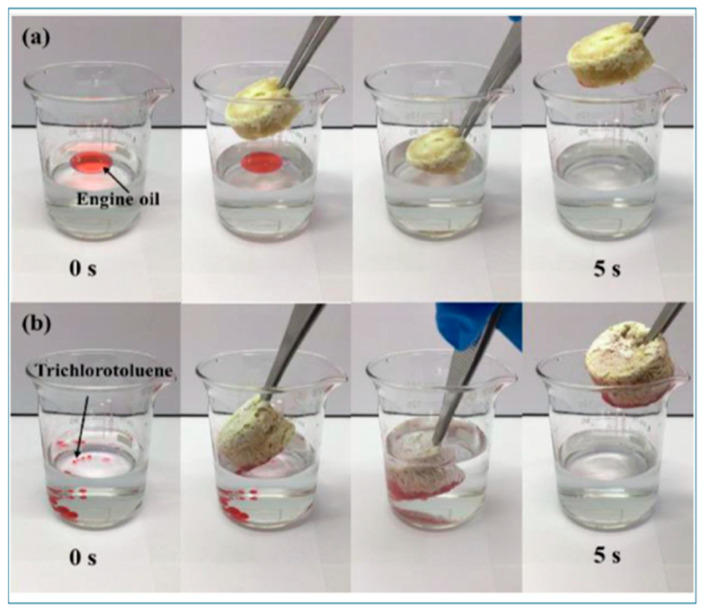
Removal of engine oil on the surface of water (**a**) and (**b**) removal of trichlorotoluene at the bottom of water by CNF-C/PEI/MBTex aerogel (engine oil and trichlorotoluene dyed red with Sudan III), demonstrating the aerogel’s ability to adsorb oils from both the surface and bottom of water bodies [143]. Copyright: image has been taken with permission from @ 2023, Elsevier.

Treatment of oil spills and oily wastewater is still a great environmental issue that has resulted in the demand for new materials that can efficiently adsorb oil [141]. Tang et al. has prepared a novel composite aerogel, synthesized from carboxylated cellulose nanofibers (CNF-C), exfoliated bentonite (BTex), and Ti_3_C_2_ MXene, as a solution to this issue [142]. The adsorption of high-viscosity crude oil by CNF-C/Ti_3_C_2_ and CNF-C/BTex/Ti_3_C_2_ aerogels was examined under ambient and photothermal heating conditions. At ambient temperature, both aerogels demonstrated sluggish oil absorption, with incomplete adsorption persisting even after 240 min, principally attributable to the high viscosity and inadequate fluidity of crude oil. When subjected to simulated solar irradiation (1 kW·m^−2^), the surface temperature of the aerogels escalated swiftly, decreasing crude oil viscosity and facilitating accelerated infiltration into the porous structure. The CNF-C/BTex/Ti_3_C_2_ aerogel exhibited significantly improved performance, attaining total adsorption in 20 s, in contrast to 120 s for CNF-C/Ti_3_C_2_. This enhancement can be described as the synergistic interaction between exfoliated bentonite and Ti_3_C_2_, which augments photothermal conversion efficiency, thermal conductivity, and surface lipophilicity [142]. Figure 13 shows comparison of crude oil adsorption by CNF-C/Ti_3_C_2_ and CNF-C/BTex/Ti_3_C_2_ aerogels under unheated (room temperature) and photothermal-heated (1 sun, 1 kW·m^−2^) conditions. Tang et al. [143] introduced a novel carboxyl cellulose nanofibers/polyethyleneimine/magnetic exfoliated bentonite (CNF-C/PEI/MBTex) aerogel for oil–water separation. The hybrid material leverages the properties of each phase: magnetic exfoliated bentonite (MBTex) provides magnetic separability, mold resistance, and mechanical rigidity, whereas polyethyleneimine (PEI) crosslinks with nano-cellulose to form a sponge-like framework and prevent corrosion of the magnetic medium. The resulting aerogel exhibited superhydrophobicity, good mechanical toughness, and a high oil adsorption capacity, making it a promising candidate for oil spill cleanup and oily wastewater treatment. The CNF-C/PEI/MBTex aerogel demonstrated an excellent oil adsorption capacity (24.6 to 77.8 times its own weight) and could be regenerated through simple hand squeezing to recover the adsorbed oil, retaining over 90% of its initial capacity after 20 cycles. Figure 14 illustrates the removal of engine oil by CNF-C/PEI/MBTex.

## 5. Challenges and Future Perspectives

Cellulose–bentonite composites are becoming popular as eco-friendly materials for cleaning up water and soil systems by removing different kinds of pollution, but there are a number of problems that make it hard to use them in real life. One big problem is that the composites do not selectively adsorb, which makes them less effective in complicated environmental matrices with a lot of different pollutants that are competing with each other. The composites’ physical and chemical stability over multiple adsorption–desorption cycles is also still an issue because structural degradation can affect performance. The process of regeneration itself often needs harsh chemicals or a lot of energy, which makes the composites less sustainable. Also, cellulose and bentonite are naturally degradable, but using synthetic crosslinkers or modifiers to prepare composites can make them less degradable and raise environmental concerns, especially when it comes to removing used adsorbents that are full of contaminants. Natural organic matter in real water bodies can also block adsorption sites, which makes performance even worse and makes the composites harder to reuse. Finally, it is hard for industries to adopt this technology because it is hard to scale up production while keeping the dispersion and performance consistent.

Even with these problems, the future looks bright for cellulose–bentonite composites in environmental cleanup, as long as the right innovations are made. Functionalization of the composites with specific chemical groups or nanomaterials can significantly enhance selectivity and adsorption capacity. Using bio-based crosslinkers and water-based processing to develop green, low energy synthesis methods will enhance the material’s alignment with sustainable development goals. Adding these composites to hybrid systems like membrane filtration units, fixed bed reactors, or catalytic platforms could make them more useful and help them work better in the real world. Mechanistic studies, along with computer modeling and machine learning tools, should help speed up the process of designing better materials in a logical way. Also, future research should focus on field scale validation to check long term usability, operational efficiency, and environmental safety. It will also be important to perform full life cycle assessments and create clear rules for businesses to follow in order to make commercialization easier and ensure that the technology is used in an environmentally responsible way.

## 6. Conclusions

Due to their biocompatibility, biodegradability, renewability, and chemical functionality, cellulose–bentonite composites represent a promising class of sustainable materials for advanced wastewater treatment. Through diverse synthesis approaches including physical blending, in situ hybridization, crosslinking, and surface functionalization, researchers have successfully developed tailored composite systems capable of targeting a broad spectrum of pollutants. The reported studies demonstrate the effectiveness of cellulose–bentonite composites in the removal of heavy metals, synthetic dyes, oils, and pesticides, and removal efficiency is higher for cellulose–bentonite composites rather than their individual material components. The adsorption efficiency is strongly influenced by physicochemical parameters such as pH, initial concentration, contact time, and composite composition. Moreover, the incorporation of nanostructures and functional groups has further enhanced their performance by increasing surface reactivity and pollutant affinity. The recyclability and regeneration potential of these composites make them attractive for sustainable water remediation processes.

Despite their potential, key challenges remain for cellulose–bentonite composites. Their long-term mechanical and chemical stability under complex wastewater matrices, process scalability, and regeneration efficiency must be addressed to facilitate their transition from laboratory prototypes to industrial-scale solutions. Further, a mechanistic understanding of pollutant–composite interactions remains a critical area for exploration, particularly through kinetic modeling, spectroscopic characterization, and advanced simulation techniques. Future directions should focus on multifunctional design strategies, integration with catalytic or photocatalytic systems, and testing under real field conditions. The development of cellulose–bentonite composites aligned with circular economy principles can pave the way toward greener, more efficient and cost-effective water treatment technologies, contributing significantly to global efforts in environmental protection and resource sustainability.

## Figures and Tables

**Table 1:** Preparation methods of cellulose–bentonite composites based on physical and chemical interactions.

Preparation Method	Type of Method	Nature of Interaction	Process	Ref.
Solution casting	Physical	Hydrogen bonding, electrostatic, van der Waals	Simple mixing and drying of aqueous dispersions; widely used for films.	[62]
Mechanical mixing/melt blending	Physical	Physical embedding, weak interactions	Thermal or shear blending; suitable for thermoplastic cellulose derivatives.	[63]
Freeze-drying (sol–gel route)	Physical	Physical gelation, hydrogen bonding	Used for aerogels and porous scaffolds; retains structure during drying.	[64]
Electrospinning	Physical	Physical entrapment, hydrogen bonding	Produces nanofibrous mats; often uses cellulose acetate.	[65]
Layer-by-layer (LbL) assembly	Physical	Electrostatic interactions, hydrogen bonding	Thin film coatings via sequential deposition; high structural control.	[66]
In situ precipitation	Physical/Chemical	Primarily physical; may involve mild chemical crosslinking	Cellulose precipitates in presence of clay; it may include pH or salt changes.	[67]
In situ polymerization	Chemical	Covalent bonding, intercalation, grafting	Polymer formed from monomers in the presence of bentonite; synthetic-based.	[35]
Chemical crosslinking/grafting	Chemical	Covalent bonding	Crosslinking agents (e.g., glutaraldehyde, citric acid) used with cellulose.	[68,69]

**Table 2:** Removal of different dyes by pure cellulose, bentonite, and cellulose–bentonite composites.

Cellulose–Bentonite Composite	Dyes Removed	Efficiency by Cellulose	Efficiency by Bentonite	Efficiency by Composite	Ref.
Bentonite/carboxymethyl cellulose-g-poly(2-(dimethylamino) ethyl methacrylate) composites	Anionic dyes (Congo red (CR) and methyl orange (MO))	<50%	~40–50%	93.50%, 87.58%	[96]
Acrylic polymer emulsion/bentonite coated on cotton cellulosic fiber (APE/bentonite–CCF, called CAC)	Brilliant green (BG) dye on CAC	70–80%	<80%	95–97%	[93]
Acrylic polymer solution (APS) mixed with bentonite (ben)	Methylene blue (MB)	<20–30%	60–85%	100%	[97]
Polyvinyl alcohol/carboxymethyl cellulose hydrogels reinforced with graphene oxide and bentonite	Methylene blue (MB)	83.33 mg g^−1^	50–119 mg g^−1^	172.14 mg g^−1^	[98]
Carbon/montmorillonite (Mt) composite (CMt)	Methylene blue (MB)	~83.3 mg g^−1^	~47.2 mg g^−1^	138.10 mg g^−1^,	[99]
Cellulose–bentonite (CB) porous composite hydrogels	Congo red	12.0 mg g^−1^	20.97 mg g^−1^	45.77 mg g^−1^	[94]
Bentonite–zeolite–acrylic polymer	Brilliant green (BG)	-	22.78 mg g^−1^	90.09 mg g^−1^	[54]
Carboxymethyl cellulose/organo-bentonite (CMC/OBent)	AR42 anionic dye	Low	negligible	29.16 mg g^−1^	[100]
Bacterial cellulose (BC) and Ca-montmorillonite (Ca-MMT) composites	Methylene blue (MB)	122.2 mg g^−1^	286 mg g^−1^	338.8 mg g^−1^	[101]
Exfoliated bentonite sheets admixed with nano-cellulose fibers (EXB/CF)	Safranin dye	93.15%	-	34 mg g^−1^	[102]
La(III)-supported Carboxymethyl cellulose–bentonite composite	Indigo carmine (IC), Acid Blue 158 (AB158) and Reactive Blue 4 (RB4)	14.4%, 16.5%, and 18.3%	24.5%, 25.3%, and 26.2%	80.41%, 83.54%, and 86.91%	[103]
Zr(IV) encapsulated carboxymethyl cellulose montmorillonite composite	Reactive red 2 (RR) and acid orange 7 (AO) dyes	less	less	97.5%, 96%	[104]
Cellulose/clay composites	Drimarine Yellow HF-3GL direct dye	less	less	89.95%	[105]
Clay/cellulose composite	Rhodamine B	less	227.27 mg g^−1^	94.7%	[106]
Polyvinyl alcohol-carboxymethyl cellulose-sodium alginate (PVA/CMC/SA)	Cationic dye (malachite green, MG)	-	-	99.12%	[107]
Ethyl cellulose (EC) filled with bentonite (Bent)	Methylene blue (MB)	70–80%	<80%	83%	[78]
Cellulose/clay/sodium alginate composites	Methylene blue (MB)	70–80%	<80%	90%	[108]
Pineapple peel cellulose/bentonite composite hydrogels	Methylene blue	70–80%	<80%	55. 87 mg g^−1^	[109]
Chitosan and Carboxymethyl cellulose-based hydrogel	Malachite green	92%	90%	96.09%	[110]
Sodium carboxymethyl cellulose-dextran sulfate and silver nanoparticle-modified zeolite (CMC-DS-AgZ)	Basic red 46 (BR46) and methylene blue (MB)	less	less	344.82 and 454.55 mg g^−1^	[111]
Cellulose-modified bentonite composite doped with iron (Fe@C/Bt)	Methyl orange	337 mg g^−1^	308 mg g^−1^	98%	[112]

**Table 3:** Removal of different metal ions by pure cellulose, bentonite, and cellulose–bentonite composites.

Composite Material	Metals Removed	Preparation Method	Removal Efficiency of Cellulose	Removal Efficiency of Bentonite	Removal Efficiency of Composite Material	Ref.
Cellulose/bentonite in NaOH/urea	Pb^2+^	Optimal extrusion dropping technology	1.318 mg g^−1^	14.71 mg g^−1^	2.86 mg g^−1^	[85,123,124]
Cellulose–bentonite/L-cystein	Cu^2+^, Pb^2+^, and Cd^2+^	Solution casting	8.98, 6.97, and 5.87 mg g^−1^	7.33, 5.09, and 4.83 mg g^−1^	32.36, 18.52, and 16.12 mg g^−1^	[60]
Magnetic Cellulose Nanocrystal/Metal	Pb^2+^	Mechanical agitation method	92.24 mg g^−1^	177.27 mg g^−1^	558.66 mg g^−1^	[125]
Poly (itaconic acid/methacrylic acid) grafted-nanocellulose/nanobentonite composite	Co^2+^	Radical polymerization	-	128.2 mg g^−1^	347.8 mg g^−1^	[126]
Carboxymethyl cellulose bentonite adsorbent	Ar^3+^	Batch adsorption studies	-	17%	9.4 mg g^−1^	[127]
Lignin xanthate resin (LXR) intercalated bentonite clay composite (LXR-BT)	Hg^2+^	Intercalation	-	119.93 mg g^−1^	438.75 mg g^−1^	[128]
Magnetic Fe_3_O_4_-chitosan@bentonite (Fe_3_O_4_-CS@BT)	Cr^+4^	Hydrothermal synthesis followed by chemical crosslinking and composite formation	Less	Less	62.1 mg g^−1^	[129]
Bacterial cellulose/chitosan composite aerogel	Cu^2+^, Cr^6+^	Facile method	-	lower efficiency	200.6 mg g^−1^, 152.1 mg g^−1^	[102]
Straw/bentonite-g-poly (acrylic acid-*co*-acrylamide)	Cd^2+^, Pb^2+^	NA	less	less	315.1 mg g^−1^, 355.5 mg g^−1^	[130]
Carboxymethyl cellulose (CMC)/Ca-montmorillonite clay composite	Cu^2+^	Blending using solvent method	less	less	54.6 mg g^−1^	[131]
Polyethylenimine and carboxymethyl cellulose co-modified magnetic bentonite	Pb^2+^, Cd^2+^	Grafting reaction	175.44 mg g^−1^	100 mg g^−1^	760 mg g^−1^, 470 mg g^−1^	[132]
Microcrystalline cellulose (MCC) and bentonite (Ben)	Cd^2+^	Grafting reaction	very low	-	242.53 mg g^−1^	[133]
CMC/SA hydrogel with CaCO_3_ nanoparticles and bentonite	Pb^2+^, Cu^2+^, and Cd^2+^	Crosslinking reaction	-	81.5 mg g^−1^,19.6 mg g^−1^, 21.7 mg g^−1^	40.13 mg g^−1^, 36.83 mg g^−1^, 35.52 mg g^−1^	[134]
Carboxymethyl cellulose/sodium alginate (CMC/SA) hydrogel beads modified with calcium carbonate (CaCO_3_) nanoparticles and bentonite (Be)	PO_4_^3−^	Crosslinking reaction	93 mg g^−1^	122.2 mg g^−1^	147.2 mg g^−1^	[135]
carboxymethyl cellulose (CMC) hydrogel modified with montmorillonite (MMT)	Ar^3+^	Free radical polymerization	Negligible	low	85%	[136]

## Data Availability

No new data were created or analyzed in this study. Data sharing is not applicable to this article.
